# Urethral Calculus in a Steer

**Published:** 1896-07

**Authors:** 


### URETHRAL CALCULUS IN A STEER.

Dr. V. P. Smith, of Jamestown, Ohio, reports the case of a yearling steer, on April 22d, suffering from severe colicky symptoms, lying on its side, moaning, kicking at its side, rising, and lying down every few minutes. Suspecting from a similar case cystic calculus, examination was made, but failed to reveal the presence of any. Having been turned to grass a few days before, I administered purgatives in conjunction with opiates and stimulants. Owner reported calf all right the following day and purging. The animal was afterward turned to grass again, but was noted not to eat much, and on May 3d was subject to another attack similar to the first; treatment same as before, but the calf died on the morning of the 5th.

*Autopsy.* Abdominal cavity contained a large quantity of fluid which proved to be urine; bladder ruptured on lower surface and wall thin; upper wall thickened and inner surface showing inflammatory lesions. The urethral canal showed the presence of a calculus in the lower curvature of the penis. Small intestine was twisted, which probably arose during the violent movements of the animal and prevented external manifestation of the action of the physic, though the contents of stomach were fluid.

